# *Astyanax hastatus* Myers, 1928 (Teleostei, Characidae): A new species complex within the genus *Astyanax*?

**DOI:** 10.1590/S1415-47572009005000055

**Published:** 2009-09-01

**Authors:** Karine Frehner Kavalco, Karina de Oliveira Brandão, Rubens Pazza, Lurdes Foresti de Almeida-Toledo

**Affiliations:** Universidade Federal de Viçosa, Campus de Rio Paranaíba, Rio Paranaíba, MGBrazil; 2 Departamento de Genética e Biologia Evolutiva, Instituto de Biociências, Universidade de São Paulo, São Paulo, SPBrazil

**Keywords:** *Astyanax hastatus*, molecular cytogenetics, karyotypic evolution, species complex

## Abstract

Four populations of *Astyanax hastatus* Myers 1928 from the Guapimirim River basin (Rio de Janeiro State) were analyzed and three distinct cytotypes identified. These cytotypes presented 2n = 50 chromosomes, with 4M+8SM+10ST+28A (Cytotype A), 8M+10SM+14ST+18A (Cytotype B), 6M+8SM+4ST+32A (Cytotype C) and scanty heterochromatin, mainly located throughout pericentromeric regions of several chromosomal pairs. No homologies with the As-51 satellite DNA were observed in the three cytotypes, although all of them presented multiple 18S rDNA sites, as detected by both silver nitrate staining and FISH (fluorescent *in situ* hybridization). The application of the term “species complex” in *Astyanax* is discussed from a cytotaxonomic viewpoint.

## Introduction

Characins comprise many species of small fishes of the genus *Astyanax.* They are widespread from the southern United States down to north Argentina (Eigenmann, 1921). This Neotropical genus is composed of nearly 90 valid species, mostly found in small bodies of water.

The data available in the literature indicate that the modal chromosomal number for the genus *Astyanax* is 2n = 50 chromosomes, although a wide variation in chromosomal constitution has often been reported. Probably, non-Robertsonian re-arrangements, such as pericentric inversions, have played a key role in the chromosomal diversity of this genus (Pazza and Kavalco, 2007).

Based on chromosomal features, three “species complexes” have been identified within *Astyanax* (Moreira-Filho and Bertollo, 1991; Fernandes and Martins-Santos, 2004; Pazza *et al.*, 2006). Despite the absence of distinguishable morphological traits within the previously mentioned *Astyanax* groups, differences in the chromosomal number and karyotype formulae, apart from other macrostructural features, are easily identified among distinct cytotypes.

Moreira-Filho and Bertollo (1991) reported karyotypic variation in populations of “*A. scabripinnis*”, comprised of variations in the diploid number (2n = 46, 48 or 50 chromosomes) and in the pattern of constitutive heterochromatin distribution. Based on these data, the authors concluded that distinct karyotypes could correspond to unique evolutionary units, since each cytotype probably arose from allopatric speciation processes.

The group “*A. fasciatus*” presented high karyotypic diversity. Two “standard” cytotypes were characterized by the exclusive presence of homologous chromosomes (bearing 2n = 46 and 2n = 48). However, other variant cytotypes were also detected with 2n = 45 and 2n = 46, besides several types with 2n = 47, all co-existing under sympatric and syntonic conditions, whereat not all the chromosomes presented their counterparts. In this case, inter-cytotypic hybridization was considered as a probable hypothesis for explaining the occurrence of variant karyotypes (Pazza *et al.*, 2006). Although a certain degree of gene flow was present among the cytotypes, readily detectable hybrids were not found, thereby indicating the occurrence of incipient divergence within the group (Pazza *et al.*, 2007).

These “species complexes” are characterized by wide cytogenetic variation, mainly regarding distinct chromosomal numbers. However, the differences between karyotypes could also be restricted to karyotype formulae, or in other words, the karyotypes might present different chromosomal types, thereby suggesting the occurrence of non-Robertsonian re-arrangements. For instance, “*A. altiparanae”* represents another quite variable group, in which FN (fundamental number) values in the reported cytotypes range from 76 to 100, whereas the diploid number (2n = 50) remains the same (Fernandes and Martins-Santos, 2004).

**Figure 1:**
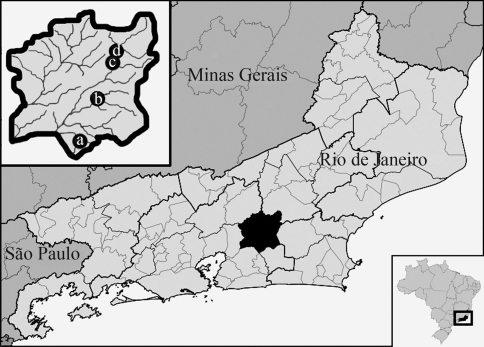
Map showing four different sites along the Guapimirim river basin, in the State of Rio de Janeiro, Brazil. In the large box, the area of Cachoeiras de Macacu county indicating sampling sites: (a) Ypiranga community; (b) Santana de Japuíba county; (c) Macacu river and (d) the town of Cachoeiras de Macacu.

In the present work, we present cytogenetic data regarding distinct populations of *Astyanax hastatus,* a species where all prior information on karyotypes is lacking. The analyzed specimens were collected in four different sites along the Guapimirim River basin (State of Rio de Janeiro) (Figure 1). Chromosomal studies involving conventional and molecular techniques were undertaken with a view to increasing current knowledge on the chromosomal evolutionary pathways in this species-rich genus. The “species complex” concept and its application to the set of distinct cytotypes of *A. hastatus* and other *Astyanax* species that also present closely related cytogenetic variant forms, are discussed.

## Material and Methods

Specimens of *A. hastatus* from four distinct localities (Table 1, Figure 1) along the Guapimirim River basin, a part of the Coastal River Basin, at Serra dos Orgãos, Rio de Janeiro, Brazil, were analyzed. The sampled specimens reached up to 10 cm in standard length, although individuals of less than 3 cm in length were more frequent. All specimens were identified and deposited in the collection of the Museum of UFRGS and MCT (PUC-RS), Brazil.

The mitotic chromosomes were obtained according to Gold *et al.* (1990). Silver nitrate staining (Ag-NOR) was done according to Kavalco and Pazza (2004). C-banding followed the procedure as described by Sumner (1972). Fluorescent *in situ* hybridization (FISH) (Pinkel *et al.*, 1986; Pazza *et al.*, 2006) was performed by using 18S rDNA probes (Hatanaka and Galetti Jr, 2004) and a satellite DNA probe (As-51) isolated from *A. scabripinnis* (Mestriner *et al.*, 2000).

Chromosomal preparations were analyzed under a light microscope and the images (resolution of 5Mp) were captured using the image analysis system CoolSnap Pro and the software Image Pro Plus (Media Cybernetics). The classification of chromosomal types was based on the arm ratio (AR), as follows: M-metacentric (AR = 1.00-1.70), SM-submetacentric (AR = 1.71-3.00), ST-subtelocentric (AR = 3.01-7.00) and A-acrocentric (AR higher than 7.00), according to Levan *et al.* (1964).

## Results

All of the populations presented the same chromosomal number, Ag-NORs and 18S rDNA sites on the short arms of ST-A chromosomes and a similar pattern of heterochromatin distribution. Nevertheless, distinct karyotypic formulae were observed, with the identification of three different cytotypes.

**Figure 2:**
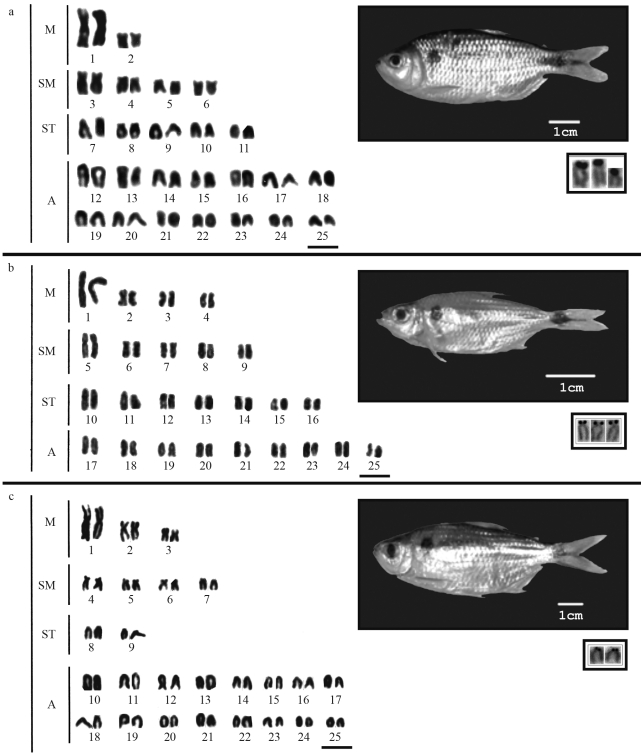
Karyotypes and specimens of cytotypes A (a), B (b) and C (c) of *Astyanax hastatus*. The respective Ag-NORs, are shown in the boxes. Bars = 5 μms.

The specimens from site (a) presented 2n = 50 chromosomes with a karyotype formula composed of 4M+8SM+10ST+28A and FN = 72 (cytotype A) (Figure 2a). The specimens from site (b) presented 2n = 50 chromosomes distributed into 8M+10SM+14ST+18A, with FN = 82 (cytotype B) (Figure 2b). Specimens from sites (c) and (d) shared a common cytotype, with 2n = 50 chromosomes arranged into 6M+8SM+4ST+32A, and FN = 68 (cytotype C) (Figure 2c).

**Figure 3:**
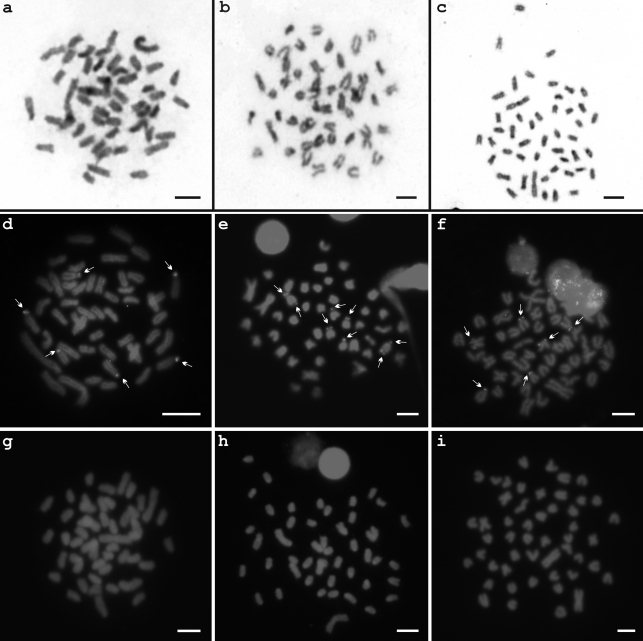
Metaphases of *Astyanax hastatus.* Cytotypes A, B and C after C-banding (a-c), and FISH with rDNA 18S (d-f) and satellite DNA As-51 (g-i) probes. The arrows indicate rDNA sites. Bars = 5 μm.

Cytotype A presented three Ag-NOR bearing chromosomes (Figure 2a – box), while six signals were identified at the terminal position of A chromosomes by 18S-FISH (Figure 3d). In the same way, cytotype B presented three Ag-NOR bearing sites (Figure 2b – box), although only four signals on the short arms of A chromosomes were observed by 18S-FISH, besides a pair bearing bitelomeric marks (Figure 3e). Cytotype C presented a variable number of silver nitrate marks, ranging from one to eight sites, with a predominance of two active NORs (Figure 2c – box). After 18S-FISH, four ribosomal DNA regions were identified at the terminal position of A chromosomes, plus a SM chromosomal pair bearing terminal marks on short arms (Figure 3f).

C-banding revealed few heterochromatic segments, mainly located at the pericentromeric region of chromosomes in all cytotypes (Figure 3a, c). No positive signals were detected after hybridization with As-51 satellite DNA probes (Figure 3g, i).

## Discussion

The genus *Astyanax* is characterized by remarkable karyotypic diversity (Pazza and Kavalco, 2007) and, according to Langecker *et al.* (1991) and Jeffery (2001), this group stands out as an excellent model for all kinds of studies on evolutionary mechanisms. In relation to Neotropical fish fauna, the genus *Astyanax* can be regarded as one of the best documented groups from a cytogenetic standpoint, with more than 60 published reports on different species. In this context, the groups *A. scabripinnis*, *A. altiparanae* (sometimes referred to as *A.bimaculatus*) and *A. fasciatus* are those that present by far the highest number of analyzed populations (for a review, see Pazza and Kavalco, 2007).

Karyotypic diversity in the genus *Astyanax* might involve diploid number and karyotypic macrostructure, the presence or absence of B chromosomes, heterochromatin polymorphism and the differential location of ribosomal sites. Due to such variability, often detected at inter- and intra-population levels, the occurrence of “species complexes” has been suggested for at least three groups within the genus (Moreira-Filho and Bertollo, 1991; Justi AJ, MSc Dissertation, Universidade Federal de São Carlos, UFSCar, Brazil.1993; Fernandes and Martins-Santos, 2004).

*A. hastatus* shares some of the features observed in the *A. altiparanae* species complex, such as a conserved diploid number (2n = 50 chromosomes), distinct karyotypic formulae and FN values (Figure 2), as well as the variable number and position of ribosomal genes. This type of variation seems to indicate that certain evolutionary processes such as pericentric and paracentric inversions might have played a key role in the chromosomal differentiation of populations of *A. hastatus*.

The ancestral karyotype within the genus *Astyanax* was most probably composed of 2n = 50 chromosomes (6M, 22SM, 10ST and 12A), since it is found at the base of neighbor-joining dendrograms based on karyotypic traits of distinct species/populations from this genus (Pazza and Kavalco, 2007). In effect, although some species of the genus *Astyanax* might present distinctive diploid numbers other than 50 chromosomes, as, for example, *A. schubarti* (2n = 36) (Daniel-Silva and Almeida-Toledo, 2005) and some populations of *A. scabripinnis* and *A. fasciatus*, which bear 2n = 46 and 2n = 48 chromosomes, respectively (Moreira-Filho and Bertollo, 1991; Justi AJ, MSc Dissertation, Universidade Federal de São Carlos, UFSCar, Brazil, 1993; Pazza *et al.*, 2006), the vast majority of the studied species do indeed present 2n = 50 chromosomes (Pazza and Kavalco, 2007).

Another feature of the genus is the presence of multiple cistrons of 18S rDNA. The 18S rDNA sites in *Astyanax* are rather dispersed throughout the karyotype, and can range either among populations of a single species or among species of a same group (Mantovani *et al.*, 2005), exactly as observed in *A. hastatus* (Figure 3d-f). They are usually observed at terminal regions of chromosomes (Ferro *et al.*, 2001; Almeida-Toledo *et al.*, 2002; Kavalco and Moreira-Filho, 2003; Mantovani *et al.*, 2005; Pazza *et al.*, 2006), or in an interstitial position (Almeida-Toledo *et al.*, 2002), and due to their reduced size, a precise determination of rDNA location and number is commonly thwarted (Ferro *et al.*, 2001; Kavalco and Moreira-Filho, 2003; Pazza *et al.*, 2006). The 18S rDNA sites in *A. hastatus* are also minute, and are located at the terminal region of NOR-bearing chromosomes (Figure 3d-f).

Although sharing these traits in common with most of the previously analyzed *Astyanax* species, the cytotype B of *A. hastatus* presented positive signals on both telomeres of an acrocentric pair after FISH experiments with 18S rDNA probes (Figure 3e). The same findings have also been reported in the genera *Hoplias* (Born and Bertollo, 2000) and *Oligosarcus* (Hattori *et al.*, 2007), as well as in *A. scabripinnis* (Malacrida *et al.*, 2003; Mantovani *et al.*, 2005) and *A. paranae* (Vicari *et al.*, 2008), the latter formerly considered as a subspecies of *A. scabripinnis*. On taking into consideration the high number of populations analyzed so far within the genus *Astyanax*, bitelomeric NORs cannot be considered as a general trend. At least for the group *A. hastatus*, they were observed in only one out of the three cytotypes described.

The As-51 satellite DNA is a repetitive DNA sequence formerly identified in *A. scabripinnis* (Mestriner *et al.*, 2000), and which has also been found in other *Astyanax* species, thereby representing a useful marker for the genus. The lack of homology with the As-51 satellite DNA observed in *A. hastatus* (Figure 3g-i) is a characteristic also reported in other species of coastal distribution, such as *A. giton*, *A. intermedius* (Kavalco *et al.*, 2007) and *A. ribeirae* (Kavalco KF, PhD Thesis, Universidade de São Paulo, Brazil, 2008). Such a satellite DNA is also absent in *A. bockmanni* (Kavalco *et al.*, 2009), a species from the upper Paraná River basin, and in a population of *A. scabripinnis* from the São Francisco River (Abel *et al.*, 2006). In relation to the species inhabiting coastal drainage systems, besides the absence of the As-51 satellite DNA, they commonly present several acrocentric chromosomes, remarkably in the karyotypes of *A. giton* and *A. intermedius* (Kavalco and Moreira-Filho, 2003), the latter displaying a karyotype that is identical to the cytotype C of *A. hastatus* (Figure 2c). More refined genetic analyses will eventually indicate whether such a resemblance represents a convergence or an ancestral feature of the group. It is worth mentioning that the species found closer to the coast along southeastern Brazil presented a higher number of A chromosomes than those located far from the shore (mainly in the Upper Paraná basin). Perhaps, this trend could reflect a vicariance process, commonly imposed on small-sized fish species (Castro, 1999). Therefore, the coastal populations might have been scattered from a single or few ancestral stocks, the subsequent gene flow constraints among sub-populations leading to their differentiation, to the point of reaching speciation. This would hypothetically explain why those *Astyanax* species inhabiting coastal areas bear several A chromosomes, whereas *Astyanax* from other drainages, as for instance *A. altiparanae*, present karyotypes with a higher number of SM chromosomes.

The expression “species complex” refers to those cases where two or more biological species are likely to co-exist, although mutual delimitation is virtually unreachable in the face of their high degree of variation (Nelson, 1999). Although characterization of species based on gene composition is hardly ever accomplishable, the variation observed through cytogenetic studies in Neotropical fishes is likely to reflect the occurrence of cryptic species rather than species complexes, since remarkable differences in chromosomes usually imply unviable crossing.

Although a correlation between organism and karyotype evolution is not always established, it seems plausible to consider that evolutionary mechanisms might have independently affected each cytotype/species through both particular environmental conditions (*i.e.*, local selective pressure) and the unique genomic features of each cytotype (karyotype formulae, heterochromatin amount and gene location). Such disruptions among distinct evolutionary levels might have been responsible for the occurrence of cryptic fish species, like those detected in *Hoplias* (Bertollo *et al.*, 1979; Dergam and Bertollo, 1990; Pazza and Julio Jr, 2003), *Eigenmannia* (Almeida-Toledo *et al.*, 1984, 1985, 1988) and, remarkably, in the genus *Astyanax*, this including *A. hastatus*. Therefore, the distinct cytotypes herein described should correspond to selection units, thus representing specific entities and composing, not a species complex, but a group of cryptic species, since each cytotype was precisely identified and the biological boundaries among analyzed specimens were supported by the cytogenetic markers used, thereby revealing the lack of hybrid forms. The same form of analysis could also be extended to other species comprising populations of different cytotypes within the genus *Astyanax*, such as *A. scabripinnis*, *A. fasciatus* and *A. altiparanae*. Although the definition of the term “species complex” might eventually undergo change, as pointed out by Nelson (1999), the terminology “cryptic species” would be suitable for those cases clearly distinguishable through cytogenetic studies.

In spite of striking chromosomal differences and the attempts at morphometric analyses through canonic variables, the subgroups within such cryptic species are not readily differentiated through morphological studies alone (Moreira-Filho and Bertollo, 1991; Maistro *et al.*, 1998; Mizoguchi and Martins-Santos, 1998b; Artoni *et al.*, 2006; Medrado *et al.*, 2008; Pazza *et al.*, 2008; among others). There are two possible explanations for this feature, one that chromosomal preceded morphological modifications, the other that these species present remarkable phenotypic plasticity. Alternatively, it could be claimed that currently performed analyses have simply failed to encounter the existing differences. Taylor (1999) stated that after the development of more sophisticated morphological analyses (*e.g.*, multivariate analysis), most cryptic or sister-species displayed a certain degree of morphological differentiation.

Based on the Darwinian concept or its revisited version (Mallet, 1995), where a species is recognized as a morphological and genotypic cluster, the cytotypes of *A. hastatus* still cannot be referred to as species in themselves. Nevertheless, according to the biological concept of a species, these cytotypes should correspond to real species (Mayr, 1969), since distinct karyotypes have already been found throughout the same hydrographic sub-basin, without any cytogenetic evidence of hybridization events.

## Figures and Tables

**Table 1:** Sample analyzed.

Locality	Collection number	GPS	N	F	M	U	Map
Ypiranga community	UFRGS 10.257	S 22° 38'11.6” Wo 42° 42'42.3”	22	8	9	5	a
Santana de Japuíba county	UFRGS 10.258	S 22° 33'39.9” Wo 42° 40'51.1”	10	2	3	5	b
Macacu river	UFRGS 10.259	S 22° 29'06.1” Wo 42° 39'40.3”	27	1	1	25	c
Cachoeiras de Macacu city	MCT 43.285	S 22° 27'51.2” Wo 42° 39'16.5”	15	9	5	1	d

N: number of analyzed specimens; F: females; M: males; U: undetermined sex.
